# No strong HLA association with MOG antibody disease in the UK population

**DOI:** 10.1002/acn3.51378

**Published:** 2021-05-15

**Authors:** Melissa Grant‐Peters, Giordani Rodrigues Dos Passos, Hing‐Yuen Yeung, Anu Jacob, Saif Huda, Maria Isabel Leite, Calliope A. Dendrou, Jacqueline Palace

**Affiliations:** ^1^ Nuffield Department of Medicine Wellcome Centre for Human Genetics University of Oxford Oxford UK; ^2^ Department of Clinical Neurology John Radcliffe Hospital Oxford UK; ^3^ Brain Institute and Department of Neurology Pontifical Catholic University of Rio Grande do Sul Porto Alegre Brazil; ^4^ Walton Centre NHS Foundation Trust Liverpool UK

## Abstract

Improvements in assays for detecting serum antibodies against myelin oligodendrocyte glycoprotein (MOG) have led to the appreciation of MOG‐antibody‐associated disease (MOGAD) as a novel disorder. However, much remains unknown about its etiology. We performed human leukocyte antigen (HLA) analysis in 82 MOGAD patients of European ancestry in the UK population. No HLA class II associations were observed, thus questioning the mechanism of anti‐MOG antibody generation. A weak protective association of HLA‐C*03:04 was observed (OR = 0.26, 95% CI = 0.10‐0.71, *p_c_* = 0.013), suggesting a need for continued efforts to better understand MOGAD genetics and pathophysiology.

## Introduction

Serum antibodies against conformational myelin oligodendrocyte glycoprotein (MOG) have recently been identified as a likely cause of acquired inflammatory demyelination in the central nervous system (CNS), distinct from multiple sclerosis (MS),^1^, ^2^, ^3^ and often referred to as MOG antibody disease (MOGAD). Clinically it may mimic aquaporin 4 (AQP4)‐antibody‐associated disease as a neuromyelitis optica (NMO) spectrum phenotype, with optic neuritis being the commonest onset and relapse presentation, but particularly in children may present as an acute demyelinating encephalomyelitis (ADEM), and more rarely as a cortical syndrome with seizures. It has been proposed that MOGAD represents a distinct and novel disease entity.^3^, ^4^, ^5^ MOGAD has a prevalence in the United Kingdom of ~2 per 100,000 individuals,^6^ has no convincing sex nor racial predominance (in contrast to MS and AQP4‐antibody disease), and can present at any age.^2^, ^3^, ^4^

Histopathological investigations of MOGAD suggest that perivenous inflammatory demyelination is characteristic, with macrophage, B, CD4^+^, and CD8^+^ T cell infiltration, and with less frequent deposition of activated complement and immunoglobulin compared to AQP4‐positive NMO spectrum disorder.^7^, ^8^ Magnetic resonance imaging characteristics of MOGAD show some overlap with AQP4‐antibody‐positive NMO spectrum disorder, but are distinct from MS.^9^ Overall, the pathology of MOGAD is very similar to that observed in the MOG‐antibody‐associated experimental autoimmune encephalomyelitis (EAE) model. In this model, high numbers of encephalitogenic T cells in conjunction with circulating MOG antibody promote an ADEM‐like phenotype, whereas an excess of MOG antibodies promotes the development of focal, confluent demyelinated lesions.^10^ Notably, EAE induction after MOG peptide immunization is dependent on major histocompatibility complex polymorphisms, which can also influence the distribution of inflammatory lesions.^11^, ^12^

Based on these findings, here we have investigated whether there is a strong effect of human leukocyte antigen (HLA) variation on MOGAD risk in individuals of European ancestry in the UK population.

## Materials and methods

All 63 patients followed in Oxford signed written consent of the NMO Tissue Bank (Oxford Research Ethics Committee C Ref: 10/H0606/56) and the audit of the MOG antibody‐positive patients was registered under the Oxford University Hospitals Trust policy. All 19 patients followed in Liverpool were consented under a study approved by the Research Ethics Service, NRES Committee London‐Hampstead (Ref: 15/LO/1433). The two sample sets were processed and genotyped together; no significant demographic, clinical or allelic differences were observed between the Oxford and Liverpool patients, and they were thus analyzed as one cohort. Control HLA allele frequencies were obtained from the Oxford BioBank (www.oxfordbiobank.org.uk), which includes 7,641 individuals aged 21–78 years from the Oxford area whose HLA alleles have been typed or imputed to typically four‐digit resolution; alleles with an imputation posterior probability of 90% or higher were included for final analysis.^13^ After excluding individuals with self‐reported immune‐related or neurological diseases, data from 6,056 individuals (49.0% females; average age of 41.51 ± 5.86 years) were analyzed. All patients and controls were of self‐reported white European descent.

### MOG antibody testing

The presence of serum MOG antibodies was tested in the Autoimmune Neurology laboratory (University of Oxford) using a cell‐based assay as described previously.^1^, ^2^ All patients in this study were positive (as opposed to low‐positive or negative) for MOG antibody and negative for AQP4 antibodies.

### HLA analysis

Patient HLA class I typing (at the *HLA‐A*, *B,* and *C* loci) was performed by Sanger sequencing‐based typing, and HLA class II typing (at the *HLA‐DRB1* and *DQB1* loci) was performed by sequence‐specific primer polymerase chain reaction at the MRC Weatherall Institute of Molecular Medicine HLA typing facility (University of Oxford). These typing methods provide intermediate resolution subtyping (typically to two or four digits). Probable HLA haplotype blocks were calculated using PHASE v2 software as previously described.^13^, ^14^

### Statistical analysis

Mann–Whitney U‐tests were used when comparing two groups for assessing differences in demographics and clinical characteristics. Fisher’s exact test (two‐tailed) was used to compare the HLA allele frequencies between patients and healthy controls. Odds ratios (ORs) were calculated using Haldane’s modification of Woolf’s method. The number of alleles tested at each locus was corrected using the Bonferroni correction (with a significance threshold of *p_c_* < 0.05).

## Results

### Demographic and disease course data

The demographics and clinical characteristics of the patient cohort are shown in Table 1, and are representative of MOGAD patients in the literature.^2^ The cohort showed a slight female preponderance of 62%, and the average age of onset was 31 years, with a broad overall onset age range of 3–69 years. The most frequent presentation was optic neuritis (42.7%), while 15.9% of patients presented with transverse myelitis, and only 1.2% had simultaneous optic neuritis and brain or brainstem attacks. Over half of the patients (59.8%) showed complete or near‐complete recovery from the onset attack, and only 7.3% showed poor recovery. Two years after onset, 37.8% of patients remained relapse‐free despite no chronic treatment, while 35.4% had relapsing disease. By 5 years after onset, the proportion of relapsing patients increased to 43.9%. More than 60% of patients who first presented with either transverse myelitis or optic neuritis and transverse myelitis remained relapse‐free at 5 years.

**Table 1:** Patient clinical characteristics

Patient cohorts	Oxford	Liverpool	Total
Patients, *n*	63	19	82
Mean age at onset ± SD (years)	31 ± 16.2	31 ± 16.8	31 ± 16.2
Pediatric cases, %	22.2	21.2	22.0
Female, %	66.7	47.4	62.2
Onset attack, %			
ON	36.5	63.2	42.7
TM	17.5	10.5	15.9
B	7.9	0.0	7.3
Simultaneous ON and TM	17.5	0.0	13.4
Simultaneous ON and B	1.6	0.0	1.2
Simultaneous TM and B	12.7	21.1	14.6
Simultaneous ON, TM and B	6.3	0.0	4.9
ADEM or ADEM‐like	11.1	26.3	14.6
Recovery from onset attack, %			
Complete or near complete	61.9	52.6	59.8
Partial	30.2	42.1	32.9
Poor	7.9	5.3	7.3
Median disease duration (range) in years	6 (2‐42)	6 (3‐18)	6 (2‐42)
Disease status at 2 years after onset, %			
Monophasic despite no chronic treatment	42.9	21.1	37.8
Monophasic with chronic treatment	19.0	5.3	15.8
Relapsing	30.2	52.6	35.4
N/A (disease duration <2 years)	7.9	21.1	11.0
Disease status at 5 years after onset, %			
Monophasic despite no chronic treatment	27.0	10.5	23.2
Monophasic with chronic treatment	7.9	5.3	7.3
Relapsing	39.7	57.9	43.9
N/A (disease duration <5 years)	25.4	26.3	25.6
Autoimmune disease co‐morbidity, %	9.5	0.0	7.3

ADEM, acute demyelinating encephalomyelitis; B, brain or brainstem; N/A, not applicable; ON, optic neuritis; SD, standard deviation; TM, transverse myelitis.

### HLA analysis of MOG‐antibody disease patients versus healthy controls

To test for HLA allele association with MOGAD, we typed HLA class I and II alleles with up to four‐digit resolution in the patient cohort and compared the data to HLA allele frequencies derived from a cohort of 6,056 healthy adult controls from the Oxford BioBank.^13^ For the HLA class I alleles, the HLA‐C*03:04 allele, which is found in 2.61% of cases and 9.12% of controls, was associated with protection against disease (OR = 0.26, 95% confidence interval [CI] = 0.10‐0.71, *p_corrected_* = 0.013; Table 2). Individual HLA‐C‐B haplotypes carrying the HLA‐C*03:04 allele, HLA‐C*03:04‐HLA‐B*40:01 and HLA‐C*03:04‐HLA‐B*15:01, were not significantly associated with disease resistance (OR = 0.32, 95% CI = 0.10‐1.00, *p_c_* > 0.05, and OR=0.28, 95% CI = 0.04‐2.04, *p_c_* > 0.05, respectively). No other HLA class I or HLA class II associations were observed (Table 3), and consideration of variables such as patient age, sex, presentation at disease onset, and disease course did not reveal any further significant associations.

**Table 2:** The frequency of HLA class I alleles in patients and controls

Allele	% of Cases (*n* = 82)		% of Controls (*n* = 6,056)	OR	95% CI (lower)	95% CI (upper)	*p*	*p_c_*
	% in Oxford (*n* = 63)	% in Liverpool (*n* = 19)						
HLA‐A								
A*01:01	18.95		19.03	0.93	0.62	1.39	0.763	1.000
	19.35	18.42						
A*02:01	25.49		27.72	0.83	0.58	1.19	0.331	1.000
	25.81	23.68						
A*03:01	16.34		14.05	1.12	0.73	1.72	0.571	1.000
	15.32	18.42						
A*11:01	7.84		6.07	1.24	0.68	2.24	0.505	1.000
	8.06	7.89						
A*29:02	5.88		3.92	1.44	0.73	2.84	0.303	1.000
	6.45	5.26						
HLA‐B								
B*07:02	17.65		14.17	1.31	0.86	1.99	0.200	1.000
	17.54	18.42						
B*08:01	14.38		14.24	1.02	0.65	1.61	0.907	1.000
	14.04	15.79						
B*15:01	3.92		5.87	0.66	0.29	1.50	0.386	1.000
	2.63	5.26						
B*40:01	1.96		5.63	0.34	0.11	1.06	0.050	0.450
	2.63	0.00						
B*44:02	9.15		10.73	0.84	0.49	1.47	0.691	1.000
	10.53	7.89						
B*44:03	7.19		5.81	1.26	0.68	2.35	0.482	1.000
	8.77	2.63						
HLA‐C								
C*03:04	**2.61**		**9.12**	**0.26**	**0.10**	**0.71**	**0.002**	**0.013**
	**3.39**	**0.00**						
C*04:01	7.19		8.96	0.77	0.42	1.43	0.481	1.000
	7.63	5.26						
C*05:01	7.19		10.35	0.66	0.36	1.22	0.231	1.000
	8.47	2.63						
C*06:02	9.80		10.23	0.93	0.55	1.59	0.895	1.000
	10.17	7.89						
C*07:01	20.26		17.80	1.14	0.77	1.70	0.527	1.000
	19.49	23.68						
C*07:02	15.69		14.89	1.04	0.67	1.61	0.821	1.000
	16.10	13.16						

The table shows alleles with a frequency of >5% in the cases or controls and with at least 90% imputation posterior probability in the control cohort. Fisher’s exact test (two‐sided) was used to calculate *p* values; these were adjusted using a Bonferroni correction (*p_c_* < 0.05). CI, confidence interval; OR, odds ratio.

**Table 3:** The frequency of HLA class II alleles in patients and controls.

Allele	% of Cases (*n* = 82)		% of Controls (*n* = 6,056)	OR	95% CI (lower)	95% CI (upper)	*p*	*p_c_*
	% in Oxford (*n* = 63)	% in Liverpool (*n* = 19)						
HLA‐DRB1								
DRB1*03:01	18.30		14.32	1.35	0.89	2.04	0.256	1.000
	15.57	21.05						
DRB1*07:01	13.07		15.01	0.86	0.53	1.38	0.435	1.000
	11.47	15.79						
DRB1*13:01	3.92		5.67	0.68	0.30	1.55	0.387	1.000
	4.10	2.63						
DRB1*15:01	15.69		14.20	1.13	0.73	1.76	0.733	1.000
	13.93	21.05						
HLA‐DQB1								
DQB1*02	28.76		24.33	1.14	0.81	1.61	0.464	1.000
	29.37	26.32						
DQB1*03:01/04	18.30		18.54	0.94	0.63	1.40	0.840	1.000
	18.25	18.42						
DQB1*03:02	13.73		9.69	1.37	0.86	2.17	0.184	1.000
	15.87	7.89						
DQB1*03:03	4.58		5.73	0.73	0.34	1.57	0.609	1.000
	3.97	5.26						
DQB1*05	14.38		15.41	0.85	0.54	1.34	0.585	1.000
	16.67	7.89						
DQB1*06:02/03	19.61		19.51	0.94	0.63	1.39	0.766	1.000
	18.25	23.68						

The table shows alleles with a frequency of >5% in the cases or controls and with at least 90% imputation posterior probability in the control cohort. Fisher’s exact test (two‐sided) was used to calculate *p* values; these were adjusted using a Bonferroni correction (*p_c_* < 0.05). CI, confidence interval; OR, odds ratio.

## Discussion

In this study, we have found possible evidence for a protective effect of the HLA‐C*03:04 allele against MOGAD, raising questions regarding the role of cytotoxic lymphocytes in the disease. Other HLA‐C*03 alleles were not associated, potentially explaining the absence of an HLA‐C*03 association in a recent study in individuals of European ancestry that employed two‐digit HLA typing in a smaller cohort of 43 patients,^15^ and high‐lighting the need for future higher resolution HLA analyses to determine if the HLA‐C*03:04 association can be replicated. HLA‐C*03:04 has also been previously reported to protect against Parkinson disease, in which neuroinflammatory mechanisms have been implicated, and against Behçet's disease and Posner‐Schlossman Syndrome.^16^, ^17^, ^18^

Despite the identification of strong HLA class II risk effects for other rare antibody‐associated CNS diseases with similar patient cohort sizes,^14^ we found no such associations for MOGAD. A recent study of MOGAD patients of Chinese Han ancestry observed an association of HLA‐DQB1*05:02–DRB1*16:02 with risk for pediatric but not adult‐onset MOGAD.^19^ These alleles have a lower frequency in populations of European ancestry and thus could not be evaluated in our study: there was only one individual in our patient cohort carrying these alleles, but this individual did have a pediatric onset of MOGAD. In a separate set of 11 individuals with self‐reported Hispanic/Latino, Black/African, Asian or mixed ethnicity, we found one individual of mixed ethnic background but with adult‐onset MOGAD who carried these alleles; none of the patients carried the HLA‐C*03:04 allele (data not shown). Considering our data in relation to the study of MOGAD patients of Chinese Han ancestry suggests that there may be population‐dependent differences in MOGAD genetic risk and etiology, but this requires further investigation.

One mechanistic interpretation of the lack of HLA class II associations is that B cell activation might occur independently of T cell help, without strict reliance on a linked recognition mechanism. A second possibility is that a number of different HLA class II‐MOG peptide complexes can stimulate CD4^+^ T cells to provide B cell help. Such heterogeneity may be consistent with the potential absence of strict tolerance establishment against MOG, as compared with other CNS antigens, given its low‐level expression in the thymus and in peripheral organs.^20^ If many different HLA class II‐MOG peptide complexes have pathogenic potential, then considering the low prevalence of MOGAD, and the emerging lack of MOG antibody in control individuals with improved antibody detection methods,^1^, ^2^ this would suggest that other factors play a key role in triggering disease and driving antibody production. A third explanation is that a MOG‐antibody‐centric disease classification, which includes patients with variable ages and presentations at disease onset, may mask underlying subgroup‐specific HLA associations. However, no further HLA associations were uncovered when considering patient subgroups, although this requires further analysis in larger patient cohorts.

Future genetic and mechanistic studies are necessary to further investigate and replicate the associations of HLA class I and II alleles and haplotypes, and to assess complement gene haplotypes and non‐HLA region variants in larger cohorts of MOGAD patients across different ethnicities, and to dissect their pathophysiological consequences.

## Author Contributions

Conception and design of the study: J.P., C.A.D. Sample and data acquisition and analysis of data: G.R.D.P., M.G.‐P., H.‐Y.Y., A.J., S.H., M.I.L., C.A.D., J.P. Drafting the text or preparing tables: C.A.D., J.P.

## Conflicts of Interest

Prof Palace reports grants and personal fees from Merck Serono, Chugai, MedImmune, Alexion, and ABIDE, and personal fees from Teva, Roche, MEDDAY, ARGENX, Mitsubishi, UCB, and Viela Bio, outside the submitted work. In addition, Prof Palace has a patent Isis: Diagnosing Multiple Sclerosis issued, and a patent Know‐how from the Numares Collaboration pending. Dr. dos Passos reports a scholarship from ECTRIMS, a scholarship from the World Federation of Neurology, grants and personal fees from Novartis and Biogen, personal fees and non‐financial support from Roche and Merck, personal fees from Bayer, and non‐financial support from Teva and Sanofi‐Genzyme, outside the submitted work. Dr. Leite is funded by the NHS National Specialized Commissioning Group for Neuromyelitis optica, UK and by the NIHR Oxford Biomedical Research Centre, UK. She has been awarded research grants from The Myaware and University of Oxford. She has received speaker honoraria or travel grants from Biogen Idec, Novartis, and the Guthy‐Jackson Charitable Foundation. Dr Leite serves on scientific or educational advisory boards for UCB, Argenx, and Viela Bio. The other authors declare no conflicts of interest.

## References

[1] Waters P , Woodhall M , O’Connor K , et al. MOG cell‐based assay detects non‐MS patients with inflammatory neurologic disease. Neurol Neuroimmunol Neuroinflamm 2015;2:e89. 25821844 10.1212/NXI.0000000000000089 PMC4370386

[2] Jurynczyk M , Messina S , Woodhall MR , et al. Clinical presentation and prognosis in MOG‐antibody disease: a UK study. Brain 2017;140:3128–3138. 29136091 10.1093/brain/awx276

[3] Reindl M , Waters P . Myelin oligodendrocyte glycoprotein antibodies in neurological disease. Nat Rev Neurol 2019;15:89–102. 30559466 10.1038/s41582-018-0112-x

[4] Cobo‐Calvo A , Ruiz A , Maillart E , et al. Clinical spectrum and prognostic value of CNS MOG autoimmunity in adults: the MOGADOR study. Neurology 2018;90:e1858–e1869. 29695592 10.1212/WNL.0000000000005560

[5] Hacohen Y , Palace J . Time to separate MOG‐Ab‐associated disease from AQP4‐Ab‐positive neuromyelitis optica spectrum disorder. Neurology 2018;90:947–948. 29695599 10.1212/WNL.0000000000005619

[6] O’Connell K , Hamilton‐Shield A , Woodhall M , et al. Prevalence and incidence of neuromyelitis optica spectrum disorder, aquaporin‐4 antibody‐positive NMOSD and MOG antibody‐positive disease in Oxfordshire, UK. J Neurol Neurosurg Psychiatry 2020;91:1126–1128. 32576617 10.1136/jnnp-2020-323158

[7] Takai Y , Misu T , Kaneko K , et al. Myelin oligodendrocyte glycoprotein antibody‐associated disease: an immunopathological study. Brain 2020;143:1431–1446. 32412053 10.1093/brain/awaa102

[8] Hoftberger R , Guo Y , Flanagan E , et al. The pathology of central nervous system inflammatory demyelinating disease accompanying myelin oligodendrocyte glycoprotein autoantibody. Acta Neuropathol 2020;139:875–892. 32048003 10.1007/s00401-020-02132-y PMC7181560

[9] Geraldes R , Ciccarelli O , Barkhof F , et al. The current role of MRI in differentiating multiple sclerosis from its imaging mimics. Nat Rev Neurol 2018;14:199–213. 29521337 10.1038/nrneurol.2018.14

[10] Lassmann H , Brunner C , Bradl M , et al. Experimental allergic encephalomyelitis: the balance between encephalitogenic T lymphocytes and demyelinating antibodies determines size and structure of demyelinated lesions. Acta Neuropathol 1988;75:566–576. 3259787 10.1007/BF00686201

[11] Weissert R , de Graaf KL , Storch MK , et al. MHC class II‐regulated central nervous system autoaggression and T cell responses in peripheral lymphoid tissues are dissociated in myelin oligodendrocyte glycoprotein‐induced experimental autoimmune encephalomyelitis. J Immunol 2001;166:7588–7599. 11390515 10.4049/jimmunol.166.12.7588

[12] Lassmann H , Bradl M . Multiple sclerosis: experimental models and reality. Acta Neuropathol 2017;133:223–244. 27766432 10.1007/s00401-016-1631-4 PMC5250666

[13] Neville MJ , Lee W , Humburg P , et al. High resolution HLA haplotyping by imputation for a British population Bioresource. Hum Immunol 2017;78:242–251. 28111166 10.1016/j.humimm.2017.01.006 PMC5367449

[14] Binks S , Varley J , Lee W , et al. Distinct HLA associations of LGI1 and CASPR2‐antibody diseases. Brain 2018;141:2263–92271. 29788256 10.1093/brain/awy109 PMC6118231

[15] Bruijstens AL , Wong YYM , van Pelt DE , et al. HLA association in MOG‐IgG– and AQP4‐IgG–related disorders of the CNS in the Dutch population. Neurol Neuroimmunol Neuroinflamm 2020;7:e702. 32198229 10.1212/NXI.0000000000000702 PMC7136059

[16] Wissemann WT , Hill‐Burns EM , Zabetian CP , et al. Association of Parkinson disease with structural and regulatory variants in the HLA region. Am J Hum Genet 2013;93:984–993. 24183452 10.1016/j.ajhg.2013.10.009 PMC3824116

[17] Mizuki N , Ohno S , Ando H , et al. HLA‐C genotyping of patients with Behçet's disease in the Japanese Population. Hum Immunol 1996;50:47–53. 8872174 10.1016/0198-8859(96)00122-x

[18] Zhao J , Zhu T , Chen W , et al. Human leukocyte antigens‐B and ‐C loci associated with Posner‐Schlossman Syndrome in a Southern Chinese population. PLoS One 2015;10:e0132179. 26161794 10.1371/journal.pone.0132179 PMC4498812

[19] Sun X , Qiu W , Wang J , et al. Myelin oligodendrocyte glycoprotein‐associated disorders are associated with HLA subtypes in a Chinese paediatric‐onset cohort. J Neurol Neurosurg Psychiatry 2020;91:733–739. 32430437 10.1136/jnnp-2019-322115 PMC7361006

[20] Handel AE , Irani SR , Hollander GA . The role of thymic tolerance in CNS autoimmune disease. Nat Rev Neurol 2018;14:723–734. 30451970 10.1038/s41582-018-0095-7

